# Viability of cultured human skin cells treated with 1,6-hexamethylene diisocyanate monomer and its oligomer isocyanurate in different culture media

**DOI:** 10.1038/s41598-021-02811-0

**Published:** 2021-12-10

**Authors:** Jayne C. Boyer, Laura W. Taylor, Leena A. Nylander-French

**Affiliations:** Department of Environmental Science and Engineering, Gillings School of Global Public Health, University of North Carolina, Chapel Hill, NC 27599 USA

**Keywords:** Cell biology, Cell death

## Abstract

The isocyanate monomer 1,6-hexamethylene diisocyanate (HDI) and one of its trimers, HDI isocyanurate, are airway and skin sensitizers contained in polyurethane paint. The toxic response of cultured skin cells to these compounds was measured by evaluating the isocyanate concentrations at which 50% of the cells die (i.e., lethal concentration 50%, LC_50_) because the relative toxicity of each form of HDI should be considered when exposure limits of HDI-based paints are set. By using a luminescent ATP-viability assay, we compared the cytotoxic effects of HDI monomer and HDI isocyanurate on cultured human skin cells (keratinocytes, fibroblasts, and melanocytes) after 4-h isocyanate exposures using culture media with varying levels of nutrients in order to also determine the effects of media composition on isocyanate toxicity. Before analysis, experimental wells were normalized to controls containing cells that were cultured with the same vehicle and media. The measured mean LC_50_ values ranged from 5 to 200 µM across the experimental conditions, in which HDI isocyanurate in protein-devoid media was the most toxic to cells, producing the lowest LC_50_ values. For HDI monomer, keratinocytes were the most resistant to its toxicity and melanocytes were the most susceptible. However, when exposed to HDI isocyanurate, the opposite was observed, with melanocytes being the most resilient and the keratinocytes and fibroblasts were more susceptible. Depending on the type of skin cells, dose–response data indicated that HDI isocyanurate was 2–6 times more toxic than HDI monomer when using protein-devoid media whereas HDI isocyanurate was 4–13 times more toxic than HDI monomer when protein-rich media was used. Therefore, if the protein-devoid saline medium alone were used for these experiments, then a significant under-estimation of their relative toxicities in protein-rich environments would have resulted. This difference is because HDI monomer toxicity was more attenuated by the presence of protein in the culture media than HDI isocyanurate toxicity. Thus, conclusions based on comparative toxicity studies and consequent inference applied to potential human toxicity can be affected by in vitro culture media conditions. The physiochemical difference in reactivity of the two forms of HDI to biological molecules most likely explains the observed toxicity differences and may have implications for skin penetration, adverse effects like skin sensitization, and systemic responses like asthma. Future studies are warranted to investigate differences in the biological availability, cellular toxicity, and immunologic sensitization mechanisms for HDI monomer and HDI isocyanurate.

## Introduction

Isocyanates are low-molecular-weight chemicals with one or more reactive N=C=O functional groups that are used to produce polyurethane products such as adhesives, spray paints, insulation, resins, sealants, foams, and surface coatings. Isocyanate exposure is a prominent cause of occupational asthma, and spray-painters who use paints that contain both monomeric and oligomeric isocyanates are at high risk of developing the condition^1–3^. The annual production of isocyanates worldwide was about 4.4 million metric tons in 2002^4^ and, currently, an estimated 440,000 workers are exposed to isocyanates in the United States^5–7^. Exposures in the general population can occur from contact with isocyanate-containing consumer goods (e.g., Gorilla glue^®^), slow-curing isocyanate coatings, materials used in housing construction, in outdoor areas near industrial sites where isocyanates are used in manufacturing, or in neighborhoods surrounding auto-refinishing businesses^8–10^. While inhalation exposure to isocyanates has been considered the most important route through which allergic sensitization may occur, animal studies have shown that skin exposure can result in respiratory sensitization^11,12^. Similarly, inhalation exposure can result in skin sensitization^13^. Evidence also exists in humans that skin exposure to isocyanates contributes to measurable isocyanate biomarker levels in workers’ plasma and urine^14–17^. Furthermore, occupational skin exposure may result in skin irritation and allergic contact dermatitis^18–20^ as well as respiratory sensitization and asthma^21–23^ even in the absence of inhalation exposure.

**Figure 1:**
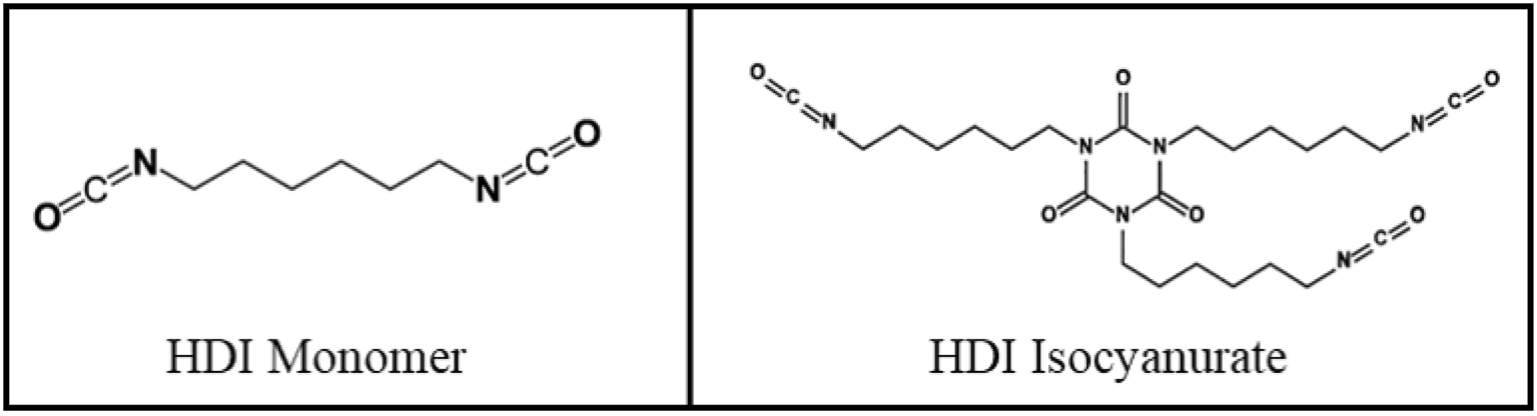
Chemical structures of HDI monomer and one of its trimers, HDI isocyanurate.

Considerable skin exposure to 1,6-hexamethylene diisocyanate (HDI) monomer and HDI isocyanurate (see chemical structures in Fig. 1) frequently occurs in the painting industry when airborne vapor, aerosols, and/or particles contact the skin via deposition, direct contact from contaminated surfaces on unprotected skin, or from unintended penetration of protective equipment^24–27^. A study using an animal model has indicated that depending upon the structure, 20–80% of isocyanates (i.e., aliphatic *vs.* aromatic, respectively) will penetrate the skin^28^. Additionally, using human skin samples we estimated that it only takes ~ 6.5 min of spray-painting for workers to achieve a dose equivalent to the American Conference of Governmental Industrial Hygienists’ time-weighted average threshold limit value (TLV^®^) of 34 mg/m^3^ for HDI monomer^29,30^. Conversely, no TLV^®^ has been set for HDI isocyanurate exposure in the workplace because less research is available for HDI oligomers. It is important to fill in the knowledge gap on HDI oligomer toxicity because HDI isocyanurate is absorbed into the human skin 350–700 times faster than the HDI monomer^30^ and HDI isocyanurate is the most abundant HDI oligomer in the automotive spray-painting environment^31^, in which spray painters’ inhalation and skin exposure to HDI isocyanurate is ≈ 300-fold greater than HDI monomer^26,27^.

Characterizing the molecular and cellular effects of isocyanates is important for understanding the consequences they have on targeted organs and systems. Skin sensitizers like isocyanates are commonly electrophilic, low-molecular-weight compounds that bind nucleophilic amino acids (e.g., cysteine and lysine) and proteins (at primary amines) causing hapten formation leading to sensitization^32–34^. Dead cell signals also activate dendritic cells and stimulate immune responses to antigens around the site of necrosis^39^ and, therefore, may increase the risk of sensitization.

The goal of this study was to investigate differences in toxicity caused by two aliphatic polyurethane precursors, HDI monomer and HDI isocyanurate, in cultured normal human skin cells. Previous studies have shown that HDI monomer, oligomers, and other isocyanates can have a variety of adverse effects on cultured cancer cell lines and animal models^13,35–42^. However, human cancer cell lines likely provide less relevant information on toxic exposure effects than normal human cells because cancer cell lines have many mutations that may cause them to respond to insults differently than do normal human cells^43^. Normal human skin cells are more challenging to culture and have a limited lifespan, but generally respond more similarly when compared to cells in vivo, and thus, their use in toxicology studies may be more informative than those of cancer cells lines.

Because the skin is one of the target tissues in occupational exposures, we compared the toxicity of the two HDI forms, HDI monomer and HDI isocyanurate, using a luminescent ATP-based cell-viability assay in normal human skin cell cultures of the three main skin cell types, keratinocytes, fibroblasts, and melanocytes. We also investigated the effect of different formulations of exposure culture media (containing varying amounts of amino acid and protein) on relative toxicity of the two isocyanates because of their known proclivity for binding primary amines. Little is known about the comparative cellular toxicity of HDI monomer versus HDI isocyanurate. It is therefore critical to determine the relative potency and dose-relationships between the HDI monomer and HDI isocyanurate exposure on the development of adverse health effects. Understanding the dose–response of skin cells to these isocyanates will help understand their relative potentials for causing adverse effects due to exposure.

## Materials and methods

### Human skin cell cultures

All methods were carried out in accordance with relevant guidelines and regulations. Skin cells were isolated from circumcised neonatal foreskin obtained from the University of North Carolina (UNC) Memorial Hospital in Chapel Hill, NC. The UNC-Chapel Hill Institutional Review Board (IRB) is the IRB for UNC Hospitals, and they determined that these skin tissues are medical waste and this study is “Not Human Subjects Research” (i.e., does not constitute human subjects research as defined under federal regulations [45 CFR 46.102 (d or f) and 21 CFR 56.102(c)(e)(l)]) and, therefore this study is exempt from needing IRB approval (IRB Exemption Study #10-1251).

The keratinocytes and melanocytes were isolated from the epidermis and fibroblasts were isolated from the dermis using a method similar to Basic Protocol 1 in “Isolation, Culture, and Transfection of Melanocytes” by Godwin et al.^44^. Fibroblasts isolated from neonatal foreskin were prepared for experiments by culturing in Dulbecco’s Modified Eagle’s Medium (DMEM; Gibco, Grand Island, NY; see Supplemental Table S1 for media components) supplemented with 10% Cosmic calf serum (HyClone; GE Healthcare Life Sciences, Logan, UT), 1X non-essential amino acids (NEAA; Gibco), and Glutamax^TM^ (Gibco). Melanocytes and keratinocytes were cultured in DermaLife Basal Medium (Lifeline Cell Technology, Frederick, MD) supplemented with LifeFactors DermaLife M or LifeFactors DermaLife K (Lifeline Cell Technology), respectively (see Supplemental Tables S2 and S3 for media components). GlutaMax^TM^ was substituted for the provided L-glutamine in LifeFactors M and K. The condition of the cultures was frequently assessed and cell cultures were passaged at a ratio of 1:3 to 1:5 for keratinocytes and melanocytes and 1:10 for fibroblasts when the cultures reached ~ 70% confluence. Cells were passaged in this fashion until they began to show signs of differentiation or senescence. For optimal culture growth, cells were allowed to recover for 24 h from thawing or passaging before use in an experiment. The medium was changed 18–24 h before plating for an experiment.

### Isocyanate treatment of skin cells

Logarithmically growing cells (~ 70% confluent) were plated in black 96-well tissue culture plates (Greiner Bio-one, Germany) at a concentration of approximately 7,000 cells per well for fibroblasts and 12,000–15,000 cells per well for keratinocytes and melanocytes. After plating, cells were allowed to attach and recover overnight prior to treatment with HDI monomer (Sigma-Aldrich, St. Louis, MO) or HDI isocyanurate (Desmodur N3300A; Bayer Material Science, Pittsburgh, PA). To prepare the isocyanate stock solutions, HDI monomer and HDI isocyanurate were initially diluted into anhydrous dimethyl sulfoxide (DMSO; Sigma-Aldrich) to concentrations of 6 M and 0.1 M, respectively. This stock was serially diluted in DMSO to various concentrations as low as 0.002 M. The individual DMSO solutions were then diluted into culture media (1:2000), and 200 µL was added to each well of cells. Isocyanate dilutions were prepared immediately before cell treatment to minimize hydrolysis. Additionally, due to the reactivity of isocyanates with amino groups, different culture media were used to test the effect of amino acids and proteins in the media on the toxicity of the isocyanates. The last step of the isocyanate stock solution dilution into culture media used cell-specific supplemented medium, cell-specific basal medium, or Hank’s Balanced Saline solution containing calcium/magnesium (HBSS+; i.e., HBSS containing calcium/magnesium to assure cell adherence during treatment) (see Supplemental Table S4 for media components). Final concentration of DMSO was 0.05% in each well. The three media types for exposure each contained different types and levels of amino acids and proteins; supplemented media contained 1.6 mg/mL of various amino acids from the basal media plus supplements with up to 7 mg/mL protein in the serum (https://www.sigmaaldrich.com/content/dam/sigma-aldrich/docs/Sigma/Product_Information_Sheet/c9676pis.pdf), the basal media contained approximately 1.6 mg/mL of various amino acids (calculated from https://www.thermofisher.com/us/en/home/technical-resources/media-formulation.8.html), and the HBSS+ is devoid of amino acids or protein. Cells were rinsed with 200 µL of culture medium immediately before treatment and then exposed to 200 µL of dilutions of either HDI monomer or HDI isocyanurate for 4 h. A 4-h exposure was used to simulate spray-painters’ maximum daily exposure time and the half-life of HDI monomer observed in our occupational studies^16,26,27^. At the end of the exposure, the medium was removed, 200 µL of supplemented growth medium was added into each well, and cells were cultured overnight to allow for cellular recovery.

### Cell viability exposure experiments

#### Cell Titer-Glo 2.0 ATP viability assay

Approximately 18 h after isocyanate exposure, the cells were rinsed with basal medium and cell viability was measured using the CellTiter-Glo^®^ 2.0 ATP luciferase-based luminescence assay according to manufacturer’s instructions^45^ using a GloMax^®^ microplate reader (Promega, Madison, WI). The ATP assay measures the disruption of membrane integrity using loss of cytoplasmic ATP as a result of endogenous ATPases and the inability to synthesize more. It is very sensitive (can detect < 10 cells per well) and is less prone to artifacts than other viability assays^46^. In this assay, the luminescent signal from reagent-lysed cells is proportional to the ATP content and, thus, is proportional to the number of live cells that are present. We included an 18-h recovery period post treatment so that the amount of ATP present was more proportional to the number of living cells, as the treatment may have inhibited ATP production but not killed some cells. Black 96-well plates were used to minimize the spill-over effect of luminescence from the adjacent wells during reading. A standard curve of rATP dissolved in the basal medium was performed to determine the linear range of the luminescence (0–6 µM rATP). The average relative light units (RLU) value from triplicate technical samples was recorded and background RLU value from the wells containing only basal medium (no cells) was subtracted from all triplicate sample RLU averages. All three media conditions were run on the same day when performing HDI monomer or HDI isocyanurate exposures. To calculate the concentrations at which 50% of the cells die (i.e., lethal concentration 50%, LC_50_), the cell viability was normalized to DMSO vehicle-exposed control cells (control wells had the same DMSO vehicle concentration and the same media as the experimental condition; supplemented, basal, or HBSS +), and then the comparative cell viability was plotted against log-transformed (base 10) exposure concentrations. The isocyanate concentration at which there was a 50% decrease in cell viability was determined from the linear portion of the curve using the linear regression equation of the line. Table 1 shows the type of skin cells used and the number of LC_50_ determinations performed with each donor’s cultured skin cells. Examples of dose–response curves for HDI monomer and HDI isocyanurate exposure in the three types of media are shown in Supplemental Materials (see Supplemental Fig. S1).

**Table 1:** Number of donors (n) and viability experiments (*N*) performed per donor for HDI monomer and HDI isocyanurate exposure.

Chemical Exposure	Cell Type	Donors	
HDI monomer	Keratinocytes Total n = 3, N = 16	Donor 1 N = 8	Donor 2 & 3 N = 4 (each)
	Fibroblasts Total n = 2, N = 11	Donor 1 N = 5	Donor 2 N = 6
	Melanocytes Total n = 2, N = 9	Donor 4 N = 5	Donor 5 N = 4
HDI isocyanurate	Keratinocytes Total n = 2, N = 10	Donor 1 N = 6	Donor 3 N = 4
	Fibroblasts Total n = 2, N = 9	Donor 1 N = 6	Donor 2 N = 3
	Melanocytes Total n = 2, N = 7	Donor 1 N = 3	Donor 4 N = 4

We used two-tailed *t*-tests to investigate differences in toxicity between exposure to HDI monomer versus HDI isocyanurate in all three cell types (e.g., keratinocyte LC_50_ for HDI monomer exposure compared to keratinocyte LC_50_ for HDI isocyanurate exposure) and to investigate cell-type specific toxicity between the given media formulations (e.g., keratinocyte LC_50_ for HDI monomer exposure in supplemented media versus in basal media). P-values were calculated using Microsoft Excel 2016 and were adjusted for multiple comparisons (a total of 27 comparisons) with R version 4.0.2 (https://cran.r-project.org/bin/windows/base/) using the p.adjust command to calculate false discovery rate (FDR) values^43^. All statistical differences were evaluated at a significance level of FDR < 0.05. The cell viability graph was created using the PROC SGPLOT command in SAS version 9.4 (https://www.sas.com/en_us/software/sas9.html).

#### Orthogonal cell viability and cell death assays

ApoTox-Glo^TM^ measures both live and dead cells concurrently using specific fluorescent substrates. Cell viability is measured via a protease detected within intact viable cells. Dead cells are concurrently quantified by detection of a dead cell-specific protease released into the medium. Cells were prepared for the assay by plating 17,000 cells per well in a 96-well plate. The test chemical concentrations used were 100 µM, 250 µM, and 750 µM HDI monomer and 1 µM, 5 µM, and 10 µM HDI isocyanurate for fibroblasts. These doses were used because they represent doses at which cell death can be detected in this assay. The toxic control, as suggested by the manufacturer of the kit, was 50 µM ionomycin in basal media. Cells were treated for 4 h. After the treatment period, 20 µL of viability or cytotoxicity reagent was added to each well, briefly mixed by orbital shaking (300–500 rpm for 30 s) and incubated at 37 °C for 45 min. Fluorescence was measured at 400 nm excitation wave-length (ex)/505 nm emission wavelength (em) (viability) and 485ex/520em (cytotoxicity) with the Cytation 3 microplate reader (BioTek, Winooski, VT).

The CellTox Green^TM^ assay (Promega) is a toxicity assay that measures cell death by detecting a fluorescent DNA intercalator that binds to DNA that is released from dead and dying cells. Since the fluorescent complex is stable, cell death can be quantified cumulatively over time. For this assay, keratinocytes were plated in a black 96-well plate at a concentration of 15,000 cells per well. Dilutions of HDI monomer (25 ﻿µM, 100 ﻿µM, 200 µM) and HDI isocyanurate (10 µM,﻿ 20 µM,﻿ 30 µM) were prepared as described above and CellTox Green^TM^ reagent was diluted into the cell-specific basal medium at a concentration of 1:1,000. Cells lysed with supplied CellTox Green^TM^ Lysis Solution acted as a 100% toxicity control. Cells were rinsed with 200 µL of test medium per well immediately before treatment and then 200 µL of diluted isocyanate containing fluorescent reagent in the cell-specific basal medium was added to each well. Fluorescence was measured every 10 to 15 min for an hour and every 20 min during the second hour. Plates were read at 485ex/520em using either a BioTek Cytation 3 (Winooski, VT) or a Promega GloMax (Madison, WI) microplate reader after an initial shaking for 30 seconds at 700 rpm. Cultures were returned to the 37 °C incubator between longer-spaced readings.

## Results

### Cell Titer-Glo 2.0 ATP viability assay

**Figure 2:**
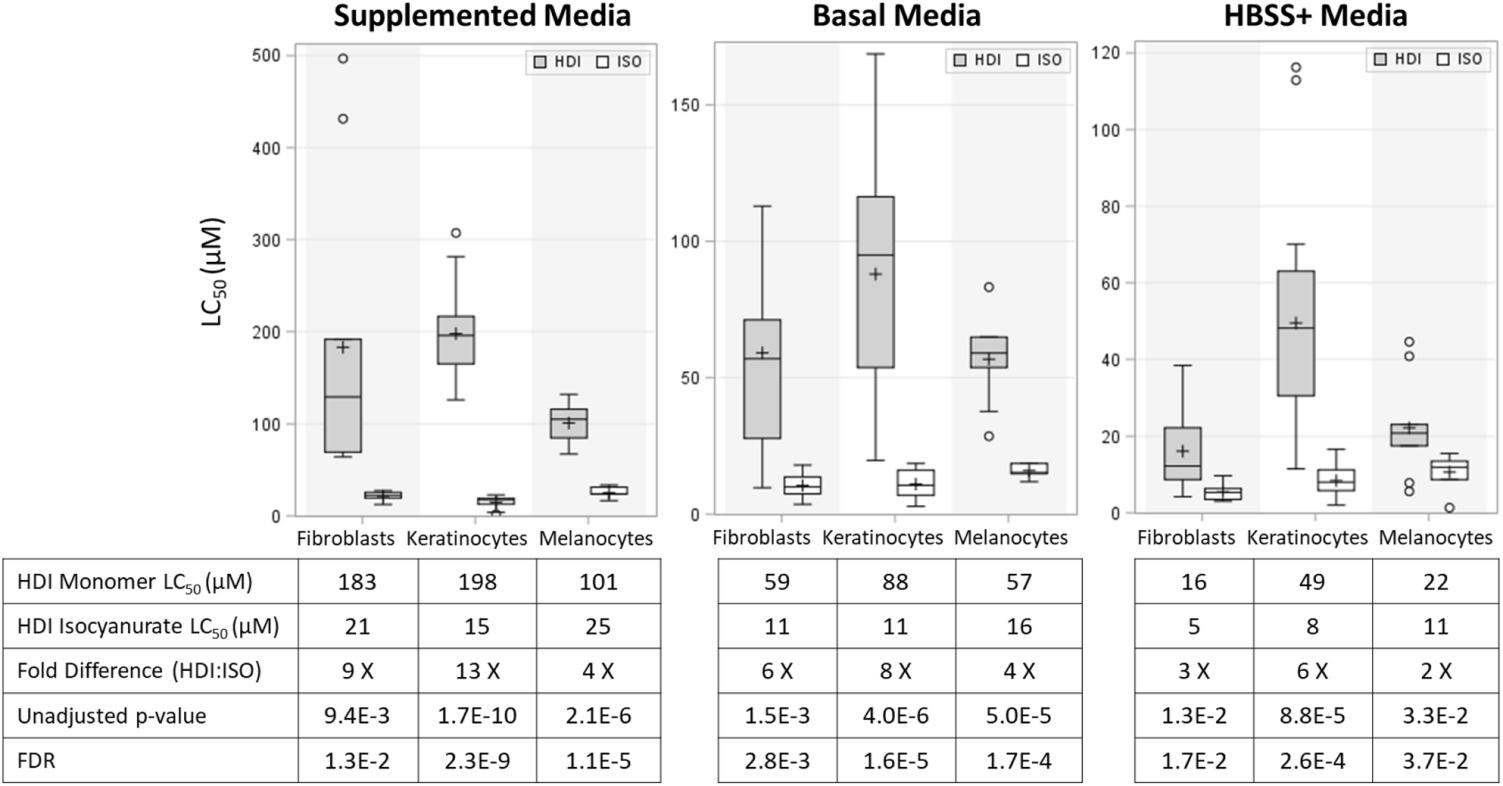
Box and whisker plots of toxicity as measured by the average concentration to kill 50% of the cells (LC_50_) for HDI monomer (HDI)- and HDI isocyanurate (ISO)-treated cultures of keratinocytes, fibroblasts, and melanocytes. LC_50_ values were determined using the CellTiter-Glo 2.0 ATP assay. Lower LC_50_ values indicate greater toxicity. Note scale differences for the different media. Median LC_50_ value (midline of the box), average LC_50_ value (+), upper and lower quartile (edges of the box), highest and lowest LC_50_ observations (whiskers), outlier LC_50_ values (o).

Using LC_50_ values calculated from CellTiter-Glo^®^ 2.0 ATP-based assay results (see Supplemental Table S5), we observed differences when investigating cell viability after HDI monomer and HDI isocyanurate exposures (see Supplemental Fig. S1 for examples of dose–response curves from which the LC_50_ values were calculated). Assessment of the relative toxicities of HDI monomer compared to its HDI isocyanurate trimer showed that HDI isocyanurate was 2–13 times more toxic than HDI monomer exposure (FDR < 0.05), depending upon the cell type and the culture media that was used during exposure (see Fig. 2). Depending on the type of skin cells, HDI isocyanurate was 2–6 times more lethal to skin cells than HDI monomer when cultured in protein-devoid HBSS+ media, was 4–8 times more lethal when using basal media with amino acids, and was 4–13 times more lethal than HDI monomer when cells were cultured in protein-rich supplemented media during the 4-h exposures (Fig. 2). Across all media, keratinocytes were the most resistant to HDI monomer toxicity and melanocytes were the most susceptible to HDI monomer toxicity (Fig. 2). However, when exposed to HDI isocyanurate, the opposite was observed. Melanocytes were the most resilient and keratinocytes were the most susceptible to HDI isocyanurate toxicity in all media except HBSS+.

Figure 2 also shows that, depending on the cell type, the LC_50_ values for exposure to HDI monomer were 16–49 μM in HBSS + protein-devoid culture media and were significantly attenuated in protein supplemented culture media requiring 101–198 μM of HDI monomer exposure to kill 50% of the cells (4–11 times less lethal, FDR < 0.05, see Table 2). The LC_50_ values for HDI isocyanurate exposure ranged from 5 to 11 μM in HBSS+ protein-devoid media and were significantly attenuated to 15–25 μM in protein supplemented media (2–4 times less lethal, FDR < 0.05, see Table 2). The LC_50_ values for the experiments performed using basal culture media with amino acids were between the values for the experiments that used HBSS+ and supplemented culture media (Fig. 2). The effect of culture media on toxicity was less pronounced for HDI isocyanurate (range 1–4-fold differences in LC_50_ values) than for HDI monomer (range 2–11 fold differences in LC_50_ values) (Table 2). Substantial differences in LC_50_ values were not observed between cells obtained from different human donors (see Supplemental Table S6). For each cell type and exposure scenario, all average LC_50_ values for the donors were within 1–2-fold of each other, except for the two fibroblast donors for HDI monomer in HBSS+ media for which there was a threefold difference in the LC_50_ values.

**Table 2:** Fold differences between the average LC_50_ values in different media for each cell type and isocyanate exposure type.

Exposure	Cell type	Supplemented : Basal (unadj p-value, FDR)	Basal : HBSS+ (unadj p-value, FDR)	Supplemented : HBSS+ (unadj p-value, FDR)
HDI monomer	Fibroblasts	3 × (3.3E−2, 3.4E−2)	4 × (3.1E−3, 5.2E−3)	11 × (8.0E−3, 1.1E−2)
HDI monomer	Keratinocytes	2 × (3.1E−7, 2.1E−6)	2 × (7.9E−3, 1.1E−2)	4 × (4.4E−11, 1.2E−9)
HDI monomer	Melanocytes	2 × (1.3E−4, 3.2E−4)	3 × (13E−4, 3.2E−4)	5 × (5.8E−8, 5.2E−7)
HDI isocyanurate	Fibroblasts	2 × (1.8E−4, 4.1E−4)	2 × (6.3E−3, 1.0E−2)	4 × (3.8E−6, 1.6E−5)
HDI isocyanurate	Keratinocytes	1 × (1.9E−1, 2.0E−1)	1 × (2.9E−1, 2.9E−1)	2 × (2.7E−2, 3.1E−2)
HDI isocyanurate	Melanocytes	2 × (1.8E−3, 3.3E−3)	2 × (2.5E−2, 3.7E−2)	2 × (2.0E−4, 4.7E−4)

### Orthogonal cell viability and cell death assays

The ApoTox-Glo^TM^ viability assay utilizes an intracellular protease to create a fluorescent signal in live cells. Ionomycin (50 µM) was used as a toxic control and exhibited ~ 37% viability after treatment in a fibroblast line. Both HDI monomer and HDI isocyanurate exhibited a dose–response using this assay (see Supplemental Fig. S2). The calculated LC_50_ values for HDI monomer and HDI isocyanurate were 1361 µM and 10 µM, respectively. These values were different than those calculated using the more sensitive CellTiter-Glo^®^ 2.0 ATP assay, however, the ApoTox-Glo^TM^ viability assay confirms that HDI isocyanurate is more toxic than HDI monomer in a dose-dependent manner. For the ApoTox-Glo^TM^ cytotoxicity assay, there appeared assay interference from the isocyanates interacting with the activity of the released cellular proteases that are the target of the assay (data not shown).

The CellTox Green^TM^ cell death assay was used to follow the dose-response of cell death from isocyanate exposure over time. This assay showed that keratinocytes exhibited a toxicity pattern to both isocyanates similar to the data using the ATP assay (see Supplemental Fig. S3), with HDI isocyanurate being more toxic than the HDI monomer. Maximal cell death occurred at about 45 minutes after exposure.

## Discussion

We characterized the in vitro cytotoxicity of two isocyanates (HDI monomer and one of its trimers, HDI isocyanurate) that are components of clearcoat polyurethane paints used in the automotive industry. We observed a significant difference in cellular toxicity between HDI monomer and HDI isocyanurate exposure using a luminescent ATP assay (CellTiter-Glo^®^ 2.0) to quantify the cell viability of three human skin cell types: keratinocytes, fibroblasts, and melanocytes. Unlike many cellular assays, the ATP assay is fast and sensitive and is not inhibited by many test compounds^46^. We observed that the HDI isocyanurate trimer was 2–13 times more toxic than the HDI monomer depending upon the skin cell type tested and the cell culture media used (Fig. 2). In addition, orthogonal cell viability and cytotoxicity assays (ApoTox-Glo^TM^ and CellTox Green^TM^) confirmed that HDI isocyanurate was more lethal to skin cells than HDI monomer. Toxicity differences between HDI monomer and HDI isocyanurate are likely due to differences in their chemical properties.

The reactivity of different isocyanates varies and characteristics such as chemical size, electronegativity, lipophilicity, permeability through biological structures, and the number of NCO groups may affect the toxicity of and biological response to isocyanates. HDI isocyanurate, which was more toxic to the cultured human skin cells, is three times larger than HDI monomer. Steric hindrance of larger structures inhibits reactions with nucleophilic compounds^47^, which would mean that HDI isocyanurate has lower reactivity than HDI monomer. Additionally, HDI isocyanurate’s NCO groups are at the end of hydrocarbon chains that are bound in the center (instead of having unreacted NCO groups on both ends of a six-carbon chain as in the HDI monomer), so its electronegativity is lower and the reaction speed of the NCO groups is likely slower than for the monomer^47^. However, the slower reactivity of oligomeric isocyanates like HDI isocyanurate may affect cellular toxicity in unexpected ways. HDI isocyanurate’s greater lipophilicity, greater number of NCO groups available for reactions (three instead of only two), and slower reaction speeds compared to HDI monomer, may all contribute to making it more likely that HDI isocyanurate will still have in-tact reactive NCO groups available for causing toxicity when it reaches cells. Future research is warranted on the difference in HDI monomer and HDI isocyanurate reactivity with nucleophilic proteins in extracellular fluids and penetration into biological membranes to shed light on their comparative in vitro toxicity.

When studying how differences in exposure culture media impact cell susceptibility, we observed that the toxicity of both isocyanates was decreased by the presence of amino acids and proteins in the media (Fig. 2), and HDI monomer toxicity was attenuated more so than was the toxicity of HDI isocyanurate (Table 2). It was not surprising that the presence of extracellular amino acids decreased the toxicity of both isocyanates. Considering that the rate with which isocyanate NCO groups react with primary amines is 1,000 times faster than the rate of hydrolysis (at 25 °C)^47^, and once the NCO groups have reacted, the resulting compound should presumably be less toxic to cells in vitro. We postulate that protein and amino acids present in media attenuate isocyanate toxicity via two mechanisms: (1) via greater resilience of the cells to toxic exposures due to greater availability of nutrients and (2) by protecting critical cellular targets from NCO reactive groups by NCO groups conjugating to amino acids and proteins in the media instead of interacting with the cells. The toxicity of HDI isocyanurate may have been less affected by the presence of proteins due to having two other NCO groups that could react with cell structures after one NCO group has reacted with amines. In contrast, the HDI monomer only has two NCO groups available for reactions instead of three. However, even in the medium without amino acids or proteins (i.e., HBSS+), HDI isocyanurate still was 2–6 times more toxic to cells than HDI monomer (Fig. 2), which could reflect the greater lipophilicity and apparent greater endogenous reactivity of this trimer compared to the HDI monomer. The significant differences observed when exposing the cells in media with different levels of nutrient supplementation indicates that it is important to conduct toxicity testing in multiple media types when evaluating chemicals that may react with media components.

When evaluating the toxicity of each chemical in the three types of exposure media (depending on the cell type tested), HDI monomer toxicity varied by 2–11-fold when varying levels of amino acids and proteins were present, whereas HDI isocyanurate toxicity only varied by 1–4-fold (Table 2). We have demonstrated that if the more sensitive protein-devoid HBSS+ medium experimental condition alone were used to predict the toxicity of HDI monomer in the protein-rich supplemented milieu, then HDI monomer’s cellular toxicity would be over-estimated by 4–11-fold, whereas HDI isocyanurate’s toxicity would only be over-estimated by 2–4-fold (Table 2, last column). When comparing the cellular toxicities of HDI isocyanurate versus HDI monomer, HDI isocyanurate was 2–6 times more toxic than HDI monomer in HBSS+ media, 4–8 times more toxic in basal media, and 4–13 times more toxic than HDI monomer when exposed in supplemented media (Fig. 1), indicating a trend of increasing difference in comparative toxicity between the two chemicals in concordance with concentration levels of amino acids and protein in the exposure media. Because of this, if only HBSS+ medium were used to assess the difference in LC_50_ values between these two isocyanates, then an under-estimation of their relative toxicity to skin cells in protein-rich environments would result.

When comparing the responses of the three different cell types (keratinocytes, fibroblasts, and melanocytes), the LC_50_ cell viability data indicated that keratinocytes were the most susceptible cell type to HDI isocyanurate toxicity in supplemented media but were the most resilient of the three cell types when exposed to HDI monomer for all three culture media (Fig. 2). Keratinocytes have been observed to be relatively resilient to toxicity, potentially because of higher enzymatic activities^48–50^. Keratinocytes are differentiated cells that make up more than 90% of the epidermis, and by differentiation and cornification, these cells produce a barrier layer to pathogens and environmental chemicals and would bear the brunt of isocyanate exposure to skin. Isocyanates are known to bind to keratin in the skin^51–53^ and their affinity for binding keratins is used in practical applications of polymer chemistry^54^. Therefore, the high abundance of protein such as keratin in these skin cells may act as a sink for isocyanate conjugation and, thus, help protect the keratinocytes themselves from isocyanate toxicity. In comparison, melanocytes were the most sensitive cell type to HDI monomer toxicity while being the most resilient to HDI isocyanurate toxicity for all media conditions (Fig. 2). Melanocytes would likely receive less exposure in vivo than keratinocytes because they reside among lower layers of keratinocytes just above the basal lamina of the epidermis and are less abundant in skin than keratinocytes and fibroblasts. For fibroblasts, intermediate susceptibility to toxicity was observed for the two isocyanates. Fibroblasts are embedded in the extracellular matrix in the dermis and their main role in wound healing makes them highly proliferative and somewhat resilient and adaptive as compared to other cell types^55^. However, the relative sensitivites of each skin cell type to isocyanate toxicity in vivo would be affected by many complex factors, and thus, in vitro toxicity studies are not fully predictive of human in vivo toxicity. Therefore, further research is needed to understand why the in vitro pattern of cell type susceptibility differs between HDI monomer and HDI isocyanurate and to investigate how that might relate to outcomes in in vivo exposure conditions. For example, because human exposure experiments would be unethical, animal models with skin structure similar to humans (e.g., procine skin) could be used to further elucidate skin cell-specific mechanisms of isocyanate toxicity.

One of the limitations of this study was the use of different media supplement compositions that were cell-specific for the supplemental media assays. There could have been a protein or amino acid target in one of the supplemented media compositions that was especially reactive with the HDI monomer (e.g., serum albumin in the fibroblast supplemental media), or there could have been notable differences in the concentrations of protein and amino acids in the cell-specific supplements. Additionally, the calf serum (10% final concentration) in the supplemented media used to culture the fibroblasts may have protected the isocyanate-exposed fibroblasts to a greater degree than the cell-specific defined media protected the melanocytes and keratinocytes, respectively. Thus, differences in the media composition may have contributed to the difference in the cell type responses to HDI monomer and HDI isocyanurate exposure in supplemented media. Although in vitro cellular toxicity information is useful, it is unknown whether toxicity is related to the sensitizing ability of isocyanates in vivo. For HDI isocyanurate, increased cell death may result in it being a more potent sensitizer. However, it is also possible that if HDI monomer haptenizes more readily to extracellular proteins, then it may increase its relative risk of immune sensitization in vivo*,* even though its interaction with amines was protective against cellular toxicity in vitro. Therefore, more studies should be carried out to evaluate differences in comparative sensitizing abilities for these two chemicals.

In conclusion, we observed a higher in vitro toxicity of cultured skin cells associated with HDI isocyanurate exposure compared to HDI monomer using two cell viability assays and one cytotoxicity assay. We also demonstrate that the toxicity was affected by media composition during the 4-h exposures. The protein-devoid media provides a better indication of the relative toxicities when there is less interference from the media, and the amino acid- and protein-supplemented media are better models of the relative cellular toxicities when the extracellular media is more complex. Notably, after normalization to control cells with the same media, HDI monomer toxicity was attenuated more by the presence of amino acids and protein in the media than HDI isocyanurate. Therefore, if the protein-devoid HBSS+ medium alone were used for these experiments, then a significant under-estimation of their relative toxicities in protein-rich environments would have resulted. The physiochemical differences in reactivity of the two forms of HDI to biological molecules most likely explains the observed toxicity difference and may have implications for skin penetration, adverse effects like skin sensitization, and systemic responses like asthma. In future studies, it is important to investigate differences in the biological availability, cellular toxicity, and immunologic sensitization mechanisms for HDI monomer and HDI isocyanurate in vivo. More informed investigations on isocyanate exposures to establish causality for associated health effects can have a significant impact on protecting the health of the general population and workers through the development of improved occupational exposure limit values for isocyanates.

## Supplementary Information

Supplementary Information.

## Data Availability

Data available upon request.
